# Effects of a 10-Week Wushu Program on Static, Dynamic, and Dual-Task Balance and Physical Fitness in Preschool Children

**DOI:** 10.3390/sports14070286

**Published:** 2026-07-07

**Authors:** Beibei Luo, Yujie Xu, Yunya Zhang, Rongda Wang, Meifeng Gu, Jingjing Wang, Yanmei Shi, Zhibei Zhou, Rui Li, Xuting Zhu

**Affiliations:** 1 School of Exercise and Health, Shanghai University of Sport, Shanghai 200438, China; 2 School of Wushu, Shanghai University of Sport, Shanghai 200438, China; 3 School of Athletic Performance, Shanghai University of Sport, Shanghai 200438, China; 4 Experimental Kindergarten Attached to Shanghai University, Shanghai 200438, China; 5 Shanghai Research Institute of Sports Science (Shanghai Anti-Doping Agency), Shanghai 200030, China

**Keywords:** children, exercise, Chinese martial arts, balance beam walk, single-leg stance, center of pressure

## Abstract

Background: As a traditional Chinese exercise, Wushu has been shown to effectively promote balance and postural stability in various populations. Preschoolers’ capacity for balance control, including static, dynamic, and dual-task balance, is linked to the later development of stability skills in adulthood. However, studies of Wushu intervention focusing on balance ability and the related physical fitness in preschool children are limited. Objectives: This study investigated the effects of a 10-week Wushu program on static, dynamic dual-task balance and physical fitness in children 5–6 years old. Methods: Seventy-three participants were randomly divided into an intervention (INT, *n* = 39) and a control (CON, *n* = 34) group. The INT group participated in a 10-week Wushu program that included three 30 min sessions per week, while the CON group engaged in unstructured free play with purposely designed materials. The three key primary outcomes were dominant-leg stance for static balance, the balance beam walk for dynamic balance, and the center of pressure (CoP) path length obtained via a force platform during dual-task balance testing, in which the participants were instructed to count numbers backward. The five derived primary outcomes were non-dominant-leg stance, CoP ML path length, CoP AP path length, CoP sway velocity, and CoP sway area. Secondary outcomes were physical fitness indicators, including sit and reach, grip strength, standing long jump (SLJ), countermovement jump (CMJ), 15 m zigzag run, double-leg continuous jump, height and body weight. The analysis of the outcomes was conducted using analysis of covariance (ANCOVA) and Pearson correlation. Results: At baseline, the CON and INT groups did not differ significantly. The INT demonstrated significant enhancements in comparison with CON in the dominant and non-dominant-leg stance (*p* = 0.017 and *p* = 0.005, respectively), the balance beam walk, SLJ, 15 m zigzag run and double-leg continuous jump (all *p* < 0.05), along with the mediolateral CoP path length (*p* = 0.012). A strong correlation was found between the balance beam walk and the double-leg continuous jump (r = 0.55, *p* = 0.001), and between the balance beam walk and the 15 m zigzag run (r = 0.43, *p* = 0.015). Conclusions: The present study demonstrates that a 10-week Wushu program significantly enhances preschool children’s static balance, dynamic balance, and mediolateral postural control during dual-task condition. The improvements in dynamic balance are correlated with lower-limb coordination and jumping agility.

## 1. Introduction

Balance abilities, including static and dynamic balance, are fundamental to children’s motor development and functional independence. Static balance represents the capacity to maintain postural stability when the body is stationary, whereas dynamic balance involves controlling posture during locomotion, running, and complex motor tasks [1]. In recent years, dual-task balance, integrating motor control with executive functions, has been recognized as a critical aspect of balance [2]. The preschool years, particularly the ages 5–6, represent a critical window for the development of balance. Specifically, postural control shifts to proprioceptive dominance at 4–5 years [3], balance at 5–6 years predicts later motor skills [4], and a sensitive period for multisensory integration occurs before age six [5]. Consequently, interventions targeting this developmental stage may yield substantial and lasting benefits.

Exercise-based interventions enhance balance performance in children, with dosage and type of exercise influencing distinct adaptations. Dynamic balance training resulted in significantly greater improvements compared to static or quasi-dynamic exercises in prepubertal children [6]. The concept of training specificity was further confirmed, demonstrating that high-difficulty training led to improvements in proactive balance, while low-difficulty training exhibited a preferential enhancement of static balance [7]. In preschool-aged children, a substantial decrease in balance beam walking time was reported following 14 weeks of integrated functional training [8]. The importance of sufficient training duration is further supported by evidence that a substantial enhancement in both static and dynamic balance was observed only after six weeks of training, with further improvements over time [9]. However, structured coordination-based and general movement programs have shown more variable outcomes. One study reported improvements only in static balance [10], while another study found no clear effects, possibly due to low training volume or insufficient dynamic stability challenges [11]. An additional study showed a non-significant advantage of structured activities over free play [12].

Therefore, the optimal development of balance in children requires progressive, dynamic, and task-specific challenges with sufficient frequency and duration. Wushu, or Chinese martial arts (Kung Fu), is well-suited to provide this form of training. A randomized controlled trial demonstrated that a martial arts program significantly improved motor skills in 5–6-year-old children [13], and a meta-analysis confirmed fitness benefits in preschool children [14]. This approach aligns with evidence that the systematic variation in task difficulty and sensory conditions optimize balance gains [7]. Moreover, Wushu incorporates sustained single-leg stances for static balance, multidirectional weight shifts for dynamic balance, and coordinated limb movements that create an implicit dual-task load. These elements provide static, dynamic, and dual-task balance challenges, a combination that is not typically found in conventional programs. Wushu exercise has been shown to enhance balance index scores in preschool children [13] and improve unipedal stability in adolescents [15]. More recent systematic reviews have corroborated that Wushu programs consistently yield gains in balance, motor competence and coordination among children and adolescents [16,17].

Although Wushu has shown benefits for balance in children, existing studies have predominantly assessed static or dynamic balance in isolation, and dual-task balance has not yet been examined in preschool children. These balance dimensions are interrelated and assessing them together may provide a more complete understanding of training effects. Furthermore, based on our previous finding that Wushu enhances lower-limb explosive power [18], we hypothesized that Wushu training improves children’s balance, potentially related to the lower-limb power and other physical fitness components. The current study therefore assessed the effects of a 10-week Wushu exercise program on static, dynamic, and dual-task balance and physical fitness among children 5–6 years of age. Static balance was assessed using dominant-leg and non-dominant-leg stances, while dynamic balance was tested using the balance beam walk. Dual-task balance was measured with a force platform. Furthermore, correlation analyses were conducted between static and dynamic balance indicators and physical fitness components related to lower-limb strength, speed, coordination, and agility. The findings may offer preliminary evidence on whether Wushu programs can address multiple dimensions of balance in preschoolers.

## 2. Materials and Methods

### 2.1. Study Design

Children were recruited from a kindergarten in Shanghai, China. All children who volunteered to participate were randomly allocated to a control (CON) group or an intervention (INT) group. Individual-level randomization was performed using a computer-generated random number sequence, and the allocation was carried out by an independent researcher who was blinded to the experimental procedures and intervention details, and who was not involved in participant assessment or intervention delivery. A two-group, repeated-measures design was adopted to assess the impact of a 10-week Wushu program on balance ability and other physical fitness–related indicators. The Wushu program was jointly developed by exercise scientists, professors in Wushu education, and kindergarten teachers. The sessions were delivered by students majoring in Wushu or Athletic Training. The free-play sessions were equipped with purposely designed materials to promote lower-limb fitness and balance, while the play itself remained child-directed. Heart rate monitoring during the sessions confirmed that the exercise intensity was comparable between the two groups. To ensure familiarity, all participating children were introduced to the full experimental protocol before any testing began. Parents or guardians were briefed on the study and gave written informed consent. Assessments were administered at baseline and on the day following the intervention, both at a consistent time, by trained Exercise and Health students blinded to group assignment to reduce bias. Each test involved standardized verbal instructions and a visual demonstration, along with one familiarization trial before the actual test. After completion of this study, children in CON were given the opportunity to learn and experience Wushu. The research protocol was approved by the Scientific Research Ethics Committee of Shanghai University of Sport (102772023RT174) and retrospectively registered in Chinese Clinical Trial Registry (ChiCTR2600127609). The CONSORT checklist is in the Supplementary File S1.

### 2.2. Participants

**Figure 1:**
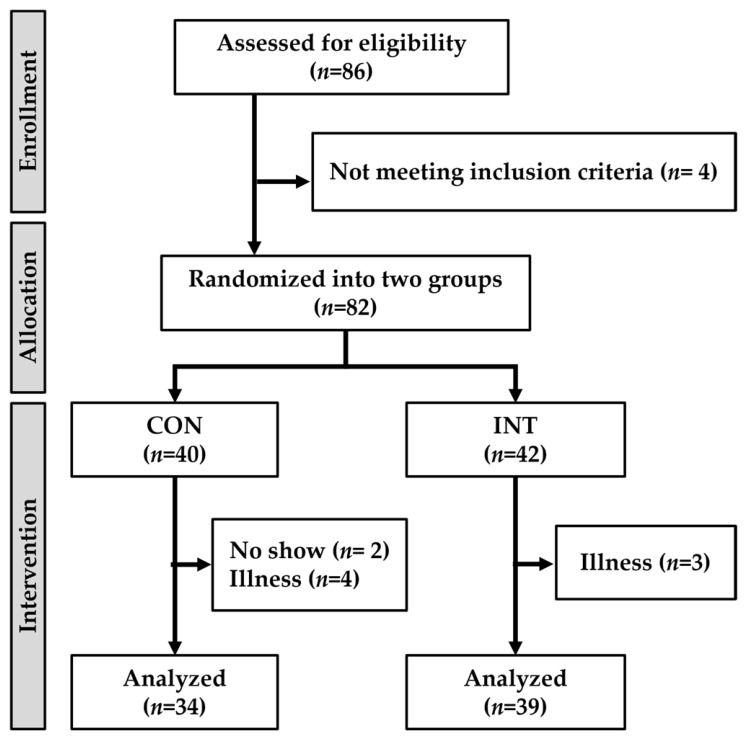
Participant flowchart.

As shown in Figure 1, 86 children aged 5–6 years were screened for eligibility. Four did not meet the inclusion criteria. The remaining 82 children were randomly assigned to the INT or CON group. Seventy-three children finished either the 10-week Wushu program (INT, *n* = 39) or the unstructured free play involving designed construction and carrying materials (CON, *n* = 34). The inclusion criteria were: (1) aged 5–6 years; (2) healthy, no recorded cardiovascular, neuromuscular, or metabolic conditions; (3) no musculoskeletal injury in the prior 6 months; (4) capable of completing all tests; and (5) written informed consent from parents or legal guardians and verbal assent from the children. The exclusion criteria were: (1) cardiovascular or respiratory diseases, or any condition making exercise unsuitable; (2) engagement in any other structured exercise training program during the study period; (3) inability to perform all tests; and (4) lack of willingness to participate by the parent/guardian or the child. The sample size was determined using G*Power 3.1.9.7. The statistical test selected was F tests (ANCOVA). The analysis was based on the following parameters: effect size f = 0.4, significance level α = 0.05, desired statistical power (1 − β) = 0.9, number of groups = 2, and number of covariates = 4 (baseline, class, age and sex). This configuration yielded a required total analyzable sample size of 68 participants (34 per group). To account for an anticipated attrition rate of around 20%, the final adjusted total sample size recruitment was increased to 86 participants.

### 2.3. Intervention

The INT group received the Wushu program from professional Wushu athletes together with kindergarten teachers. Before the intervention, a workshop and information session were held for participating parents and teachers, covering the program’s overall aim, curriculum, key precautions, anticipated benefits, and risk management. This study used a Taolu-based Wushu program. As defined by the International Wushu Federation, Taolu is a continuous sequence of predetermined techniques integrating hand strikes, kicks, jumps, sweeps, stances, footwork, and balances [19]. The program, named “Zodiac Animal Kung Fu Boogie,” consisted of twelve animal-themed movements based on the Chinese zodiac (Supplementary File S2).

The twelve movements were as follows. The Rat, a half squat, required alternating diagonal steps forward and backward, each step descending rapidly into a half squat while executing a forward thrust punch. The Ox, a horse stance, involved lateral stepping to the left and right into a wide horse-riding stance with a simultaneous thrust punch. The Tiger, performed from a high lunge, combined lateral stepping left and right with a trunk pivot and the formation of tiger claw hand postures. The Rabbit, a vertical jump, consisted of single-leg hopping on the left and right legs in place, concluding with both hands forming rabbit fist gestures in front of the chest. The Dragon, a deep lunge, integrated lateral steps left and right into a forward lunge, sinking into a low squat and simultaneously shaping dragon claw hands. The Snake, executed from a kneeling stance, began with a lateral step, descended into a single-leg kneeling squat, and extended the arms forward in a snake hand configuration. The Horse, another half squat variant, imitated a horse-riding posture by performing a single-leg standing squat while maintaining the non-supporting leg in a lifted, bent position. The Goat, a rotational jump, required a lateral rotational jump to the left and right, landing in a high horse stance and following with a pivot. The Monkey, initiated with a forward step, combined a rear back kick with the formation of a monkey fist hand gesture. The Rooster, a single-leg stance, required balancing on one leg with arms extended sideways to imitate a rooster spreading its wings. The Dog, a drag step, merged a kneeling position on one knee with a turn and a back kick, emphasizing weight transfer and stability. The Pig, a high horse stance, was a static hold in which the practitioner assumed a high horse-riding posture while holding an imaginary Tai Chi ball in front of the body, engaging the thighs and core throughout.

The objective of its design was to enhance lower-limb muscular fitness including balance through a playful and engaging approach. Basic hand gestures, lower-body stances, trunk rotations, and multidirectional jumps were integrated into all movements. Throughout the program, instructors were responsible for leading all movements, and children were not required to memorize the sequences. Each 30 min session was conducted three times per week for 10 weeks and was composed of a warm-up, Wushu practice, and a cool-down. The weekly volume for each movement was systematically varied across low, moderate, and high intensity levels. Low-intensity exercise was defined as three sets of one repetition, moderate intensity as three sets of two repetitions, and high intensity as three sets of three repetitions. As the intervention progressed, there was a gradual increase in the movement precision requirements. The instructors provided encouragement to the children.

The CON group participated in unstructured free play, with activities developed by exercise specialists to enhance lower-limb muscular strength and balance. The setting was furnished with various materials such as rollers, ladders, boxes, building blocks, and tires. These materials facilitated children’s locomotion, hopping, and standing on unstable surfaces. Heart rate was recorded for both groups during three randomly chosen sessions with an optical heart rate sensor (Polar Verity Sense, Kempele, Finland). Maximum heart rate (HRmax) was calculated as 208 − 0.7 × age. Intensity was classified as moderate when heart rate was between 64% and 76% of HRmax, and as vigorous when it was between 77% and 95% of HRmax. The intensity of exercise program was moderate-to-vigorous and comparable between the two groups.

### 2.4. Testing Procedures

#### 2.4.1. Primary Outcome

The primary outcomes were classified into three key primary outcomes and five derived primary outcomes. The three key primary outcomes were the dominant-leg stance for static balance, the balance beam walk for dynamic balance, and the total CoP path length during dual-task balance. The five derived primary outcomes were the non-dominant-leg stance, the CoP ML path length, the CoP AP path length, the CoP sway velocity, and the CoP sway area. Before each test, the procedure was explained and demonstrated to the children and their teachers, and the children performed the tasks as instructed.

Static balance was assessed using the eyes-open unilateral stance test. The experimenters performed the test on the dominant and non-dominant legs separately. The dominant leg was defined as the leg used to kick a ball. Participants were instructed to stand on a firm, flat surface, gazing at a wall target positioned at eye level. They were then asked to maintain single-leg balance for as long as possible, with a maximum time limit of 60 s. Stance time was recorded in seconds using a stopwatch. Dynamic balance was evaluated using the balance beam walk test. The subjects walked along a wooden beam (3 m × 10 cm × 30 cm) and walk time was recorded with a stopwatch. All stopwatch-based measurements were recorded by two independent trained raters, with typical agreement within 0.5 s.

For the dual-task balance test, participants stood barefoot on a force platform (KWYP-FP6035-7K, Kunwei, Changzhou, China), feet shoulder-width apart, gaze directed at a distant point. In each 30 s trial, the participants concurrently performed a cognitive task, which involved counting backwards by two digits from a starting number randomly assigned between 30 and 50. The task was administered by the same researcher. The center of pressure (CoP) was obtained from the triaxial force and moment data. Total CoP path length was obtained by summing the distances between consecutive CoP positions. Path lengths in the anteroposterior (AP) and mediolateral (ML) directions were computed as displacements along each axis. Sway velocity was derived by dividing total path length by the trial duration. The sway area was defined as the area of the 95% confidence ellipse fitted to the CoP trajectory. Each child completed two trials for all tests, and the best performance was recorded.

#### 2.4.2. Secondary Outcomes

The secondary outcomes encompassed assessments of leg and back flexibility, upper- and lower-limb strength, coordination, speed and agility, and morphological indicators. The flexibility of the subjects was assessed using the sit and reach test. The upper limb strength was measured via the handgrip test. The lower-limb strength was evaluated through the standing long jump (SLJ) and the countermovement jump (CMJ). For the SLJ, children were instructed to jump forward, extending their hips, knees, and ankles to land on both feet. For the CMJ, following a rapid downward countermovement, children jumped vertically with maximal effort. The SLJ distance and CMJ height were measured using a ruler. Coordination was assessed using the double-leg continuous jump [6], in which children executed 10 consecutive two-footed jumps across soft blocks arranged at 50 cm intervals, with the start line placed 20 cm before the first block and the finish line 20 cm after the last block. Speed and agility were evaluated by a 15 m zigzag run [6]. In this test, the subjects sprinted while weaving through 7 cones positioned at 3, 4.5, 5, 7.5, 9, 10.5, and 12 m from the starting line. Time was recorded using a stopwatch by two independent trained raters, and their values typically agreed within 0.5 s. For each test, two attempts were made, and the best result was recorded. Height and body mass were also measured, and re-measurement in three randomly selected children showed discrepancies within 0.5 cm. Body mass index (BMI) was the body mass divided by height squared.

### 2.5. Statistical Analysis

Statistical analyses were performed with R 4.2 version (R Foundation for Statistical Computing, Vienna, Austria). Frequencies and percentages were calculated for categorical variables (age, sex), and Pearson’s Chi-squared test was applied for between-group comparisons. The Shapiro–Wilk test served to check the normality of continuous variables. Mean ± SD was used to present normally distributed data, whereas median (interquartile range, IQR) was reported for non-normally distributed data. Welch’s two-sample *t*-test was used for normal data with unequal variances, and the Mann–Whitney U test for non-normal data. The efficacy of the Wushu program was evaluated by analysis of covariance (ANCOVA), with age, sex, class, and baseline pre-test values as covariates. To control multiplicity, a prespecified stratified strategy was used where the three key primary outcomes (Balance Beam Walk, Dominant-leg Stance, and CoP Path Length) were tested without adjustment. The five derived primary outcomes (Non-dominant leg Stance, CoP ML Path Length, CoP AP Path Length, CoP Sway Velocity, and CoP Sway Area) were adjusted within their family using the Holm–Bonferroni procedure, and the nine secondary outcomes were adjusted using the Benjamini–Hochberg false discovery rate (FDR) method. Significance was defined as *p* < 0.05 for the key primary outcomes and adjusted *p* < 0.05 for the derived primary and secondary outcomes. Relationships between the INT group’s post-test outcomes were explored using Pearson correlations.

## 3. Results

Seventy-three participants, aged 5.16 ± 0.37 years, achieved over 85% attendance in the 10-week Wushu program or free play. At baseline, the CON and INT groups exhibited comparable characteristics across all anthropometric and physical fitness measures (all *p* > 0.05, Table 1). The primary balance outcomes, the static balance assessed by non-dominant leg stance (*p* = 0.489) and dominant leg stance (*p* = 0.969), dynamic balance measured by the balance beam walk (*p* = 0.565), and all CoP parameters recorded during the dual task balance test, including sway velocity (*p* = 0.715), sway area (*p* = 0.586), ML path length (*p* = 0.152), and AP path length (*p* = 0.076). Similarly, the height (*p* = 0.226), body mass (*p* = 0.056), BMI (*p* = 0.24), grip strength (*p* = 0.93), sit and reach (*p* = 0.641), SLJ (*p* = 0.194), CMJ (*p* = 0.364), 15 m zigzag run (*p* = 0.122) and double-leg continuous jump (*p* = 0.25) were all comparable.

Following ANCOVA adjustment for baseline values, sex, class, and age, the INT group demonstrated significantly greater improvements than the CON group across multiple primary and secondary outcomes (Table 2). For primary balance measures, stance time increased by 7.72 s (95% CI 1.40 to 14.04, p= 0.017, η^2^p = 0.082) on the dominant-leg and by 10.73 s (95% CI 4.36 to 17.11, p= 0.005, η^2^p = 0.144) on the non-dominant-leg. Balance beam walk time decreased by 1.15 s (95% CI −1.72 to −0.58, p < 0.001, η^2^p = 0.196), indicating improved performance. Among the CoP parameters recorded during the dual-task balance experiment, only the CoP ML path length showed a significant between-group difference, decreasing by 14.22 cm (95% CI −23.42 to −5.03, p= 0.012, η^2^p = 0.124). No significant differences were found for sway velocity, sway area, total path length, or AP path length. For the secondary physical fitness outcomes, the INT group exhibited significantly greater gains in the SLJ (increase of 6.98 cm, 95% CI 3.82 to 10.15, p = 0.009, η^2^p = 0.225), Performance also improved in the double-leg continuous jump (decrease of 1.06 s, 95% CI −1.37 to −0.76, p = 0.009, η^2^p = 0.416) and the 15 m zigzag run (decrease of 0.66 s, 95% CI −0.88 to −0.44, p = 0.009, η^2^p = 0.345). No significant differences were identified for changes in height, body mass, BMI, CMJ, grip strength, or sit and reach flexibility.

**Figure 2:**
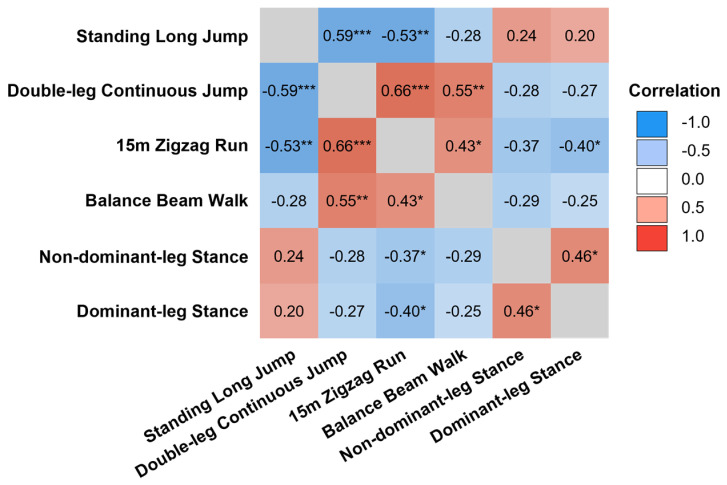
Correlation between balance and physical fitness-related indicators. Data from the post-intervention INT group. The heatmap illustrates correlation strength, with red indicating positive correlations and blue negative correlations. *: *p* < 0.05; **: *p* < 0.01; ***: *p* < 0.005.

As shown in Figure 2 and Supplementary File S3, post-intervention data from the INT group were analyzed using Pearson correlations, revealing a moderate positive correlation between the balance beam walk and the double leg continuous jump (*r* = 0.552, *p* = 0.001), indicating an association between dynamic balance and lower-limb coordination. A similar moderate positive correlation was found with the 15 m zigzag run (*r* = 0.433, *p* = 0.015), indicating an association between dynamic balance and agility. However, no statistically significant correlations were observed with the SLJ or either static balance measure (all *p* > 0.05). In contrast, both non dominant leg stance (*r* = −0.368, *p* = 0.040) and dominant leg stance (*r* = −0.399, *p* = 0.026) exhibited moderate negative correlations with the 15 m zigzag run, indicating that superior static balance is associated with faster agility performance. These static balance measures did not correlate significantly with SLJ, double-leg continuous jump, or balance beam walk (all *p* > 0.05). A moderate positive intercorrelation was demonstrated between the two static balance measures (r = 0.458, *p* = 0.010).

## 4. Discussion

The present study demonstrates that a 10-week Wushu program significantly enhances balance and physical fitness in children. Specifically, participants exhibited marked improvements in static postural control, as evidenced by increased stance duration on both dominant and non-dominant legs. These enhancements were further demonstrated by improved dynamic balance, reflected by faster completion times on the balance beam walk. The intervention also resulted in a selective reduction in the CoP ML path length during dual-task standing. Meanwhile, substantial gains were observed in lower-limb explosive power, as measured by SLJ, along with agility and coordination demonstrated by the double-leg continuous jump and 15 m zigzag run. Furthermore, dynamic balance exhibited a correlation with lower-limb coordination and agility. However, a statistically significant relationship was not identified between dynamic and static balance, given their functional independence.

CoP parameters derived from force platforms, force plates, and balance boards constitute well-validated, objective metrics for quantifying postural control in pediatric populations. Developmental studies have shown that CoP velocity and sway areas decrease with age on both rigid and foam surfaces, indicating the maturation of static balance in preschool children [20]. These studies also report that young children exhibit smaller maximum excursions in both AP and ML directions, with AP control maturing earlier than ML control [21]. Our findings show that a 10-week Wushu intervention specifically improved ML CoP path length. These results align with intervention studies in healthy children, which have demonstrated that structured exercise can enhance postural control. For instance, Obuz and Topcu reported that Pilates ball exercises enhanced static balance and dual-task performance in kindergarten children, implying improved CoP control [22]. Schaefer et al. used an ankle-disk balance board to record CoP-based body sway and found that children, unlike adults, reduced sway under dual-task conditions, prioritizing postural stability [23]. This latter finding demonstrates that CoP assessment within a dual-task paradigm can reveal child-specific attentional strategies. Our observation of improved ML path length is particularly noteworthy given that the ML axis matures later in childhood than the AP axis [21], suggesting that Wushu training may selectively target the still-developing lateral postural control system. However, the non-significant changes in AP sway and other CoP variables suggest a more complex pattern. The Wushu program was designed to be comprehensive, incorporating multi-directional movements including mediolateral weight transfer. In contrast, the intensity-matched free play of the control group naturally consisted more of forward–backward running and chasing, which primarily challenged AP postural control. Consequently, the control group may have developed AP dual-task balance comparable to that of the intervention group, thereby reducing the between-group difference in the AP axis. Additionally, the earlier maturation of AP control and the greater attentional demand on ML regulation under dual-task conditions may have further contributed to this differential pattern. Given that force platform studies in healthy preschool children remain limited, with most related work focusing on pediatric rehabilitation, future research should further explore these mechanisms.

Research on balance ability in early childhood consistently identifies age, sex, and environmental factors as major influences. Our intervention-based findings both align with and extend these observations. In China, multiple studies show that balance exhibits significant enhancements during the preschool years [3,24,25]. Our intervention significantly enhanced both static and dynamic balance, evidenced by increased single-leg stance time on the dominant leg (+7.72 s, *p* = 0.017) and non-dominant leg (+10.73 s, *p* = 0.001) during static balance assessments. Additionally, a reduction in balance beam walk time was observed, indicating enhanced dynamic balance (−1.15 s, *p* < 0.001). These findings align with and extend existing literature by demonstrating that structured exercise can effectively amplify natural developments. Zhao et al. identified a network connecting balance, body shape, and physical fitness in preschoolers [3]. Consistently, our physical fitness assessments showed concurrent improvements in explosive power (SLJ: +6.98 cm, *p* = 0.009), agility (15 m zigzag run: −0.66 s, *p* = 0.009), and coordination (double leg continuous jump: −1.06 s, *p* = 0.009). These findings suggest that the intervention may have enhanced stability not only through task-specific practice but also by strengthening underlying fitness components such as muscular power and motor coordination. The functional relevance of such integrated programming is underscored by international evidence indicating that approximately 60% of UK children aged 4–5 lack proficiency in basic stability skills [26], highlighting the value of programs that integrate balance and fitness training during early childhood.

To further analyze the possible link between balance and physical fitness, we performed correlation analyses on the indicators from the post-intervention INT group. The present findings demonstrate that dynamic balance, as measured by the balance beam walk, exhibits a significant positive correlation with lower-limb coordination and agility, evidenced by correlations with 15 m zigzag run and double-leg continuous jump. The present result aligns with intervention studies showing that coordination-based or mind–body programs enhance both dynamic balance and explosive or speed-related motor outcomes in preschoolers [11]. Although lower-limb strength has been associated with balance in children [27], no significant correlation was observed between dynamic balance and the SLJ, whereas a significant link emerged with the continuous jump. This pattern of associations suggests that dynamic stability may be more closely related to multi-joint coordination and movement velocity than to maximal leg strength alone, an interpretation supported by evidence that comprehensive physical activity interventions concurrently improve jumping, agility, and balance beam performance [28]. Strength-focused programs have been shown to improve SLJ without concomitant gains in static single-leg stance [29], whereas coordination training enhanced dynamic balance but not static balance [11]. Critically, no significant association was found between the balance beam walk and single-leg stance duration, corroborating the independence of static and dynamic balance and their potentially separate development [25,30]. A noteworthy finding was the negative correlation between static balance and agility, where better static stability was associated with faster zigzag run times. This divergent pattern indicates an association between static balance and agility, which may reflect the contribution of a stable postural foundation during rapid directional changes, a mechanism fundamentally distinct from the active postural control required during dynamic balance tasks.

This study has several limitations. First, the CON constituted an active comparison group, comprising expert-designed tasks targeting balance, locomotion, and lower-limb fitness. This active nature likely attenuated between-group differences, yielding a conservative estimate of the true Wushu training effect. Second, the absence of long-term follow-up precludes evaluation of intervention durability, and recruitment from a single educational setting may limit the generalizability of our findings. Third, cognitive performance during the dual-task balance test was recorded without systematic accuracy scoring, thereby limiting the completeness of the dual-task performance assessment. Although we implemented procedural controls such as double-rater timing and retention of the best trial, test–retest reliability was not formally assessed, which may introduce potential measurement error. Additionally, the three key primary outcomes were tested without multiplicity adjustment, and the exploratory correlation analyses were not corrected for multiple comparisons, which could inflate type I error for those analyses. Future research should incorporate long-term follow-up, multi-site sampling, accuracy metrics for cognitive tasks, and formal reliability testing. Given the distinct demands of dynamic and static balance, trials comparing static, dynamic, and combined balance protocols are warranted, with mediation analyses testing coordination as a mechanism for dynamic balance gains. Such studies could clarify whether interventions emphasizing coordination and agility for dynamic stability, or postural control and mind–body practices for static balance, yield additive or synergistic benefits for young children’s motor competence.

## 5. Conclusions

In conclusion, this study establishes that a 10-week Wushu intervention yields significant improvements in static balance, dynamic balance, and mediolateral postural control during a dual-task condition among preschool children aged 5–6 years. Concurrently, gains in dynamic balance but not static balance are positively associated with enhanced lower-limb coordination and jumping agility, reflecting the interdependent nature of these motor competencies.

## Figures and Tables

**Table 1:** Baseline characteristics.

	CON (*n* = 34) ^a^	INT (*n* = 39) ^a^	Statistic	*p*-Value
Age			χ^2^ = 0.07	0.795 ^b^
5 (yrs)	28 (82.4%)	33 (84.6%)		
6 (yrs)	6 (17.6%)	6 (15.4%)		
Sex			χ^2^ = 0.93	0.334 ^b^
female	16 (47.1%)	14 (35.9%)		
male	18 (52.9%)	25 (64.1%)		
Height (cm)	115.3 ± 4.2	116.5 ± 4.3	t = −1.22	0.226 ^c^
Body mass (kg)	20.65 (18.60, 22.00)	22.00 (19.40, 24.10)	W = 490.00	0.056 ^d^
BMI (kg/m^2^)	15.40 (14.57, 16.32)	15.72 (14.49, 17.79)	W = 556.00	0.24 ^d^
Dominant-leg Stance (s)	24 (16, 35)	22 (15, 39)	W = 659.00	0.969 ^d^
Non-dominant-leg Stance (s)	21 (9, 37)	24 (10, 45)	W = 600.00	0.489 ^d^
Balance Beam Walk (s)	5.7 (4.1, 7.4)	5.0 (4.2, 7.0)	W = 715.50	0.565 ^d^
CoP Path Length (cm)	46 (38, 54)	51 (41, 76)	W = 515.50	0.104 ^d^
CoP AP Path Length (cm)	31 (27, 37)	36 (28, 47)	W = 502.00	0.076 ^d^
CoP ML Path Length (cm)	22 (17, 27)	25 (18, 40)	W = 533.00	0.152 ^d^
CoP Sway Velocity (cm/s)	2.99 (2.51, 3.62)	3.02 (2.39, 4.13)	W = 629.50	0.715 ^d^
CoP Sway Area (cm^2^)	4 (2, 11)	5 (2, 10)	W = 613.00	0.586 ^d^
Grip strength (kg)	8.25 (5.50, 10.50)	10.00 (8.00, 10.50)	W = 511.50	0.093 ^d^
Sit-and-Reach (cm)	10.2 ± 5.9	9.6 ± 4.9	t = 0.47	0.641 ^c^
SLJ (cm)	91 ± 22	97 ± 17	t = −1.31	0.194 ^c^
CMJ (cm)	13.5 ± 4.6	14.5 ± 4.8	t = −0.91	0.364 ^c^
Double-leg Continuous Jump (s)	5.81 (5.25, 7.38)	6.41 (5.49, 7.82)	W = 558.50	0.25 ^d^
15 m Zigzag Run (s)	7.21 (6.62, 7.88)	6.87 (6.56, 7.29)	W = 803.50	0.122 ^d^

^a^: *n* (%); Mean ± SD; Median (Q1, Q3); ^b^: Pearson’s Chi-squared test; ^c^: Welch Two Sample *t*-test; ^d^: Wilcoxon rank sum test. Yrs: years. m: meters. cm: centimeters. kg: kilograms. s: seconds.

**Table 2:** Wushu Intervention effects on efficacy outcomes.

	Change from Baseline ^a^		LS Mean of Change ^b,c^		LS Mean Difference	*p*-Value ^d^	*η* ^2^ *p*
Outcomes	CON (*n* = 34)	INT (*n* = 39)	CON (*n* = 34)	INT (*n* = 39)	(95% CI) ^c^		
Dominant-leg Stance (s)	9.2 ± 13.69	13.7 ± 13.64	8.87 ± 2.55	16.59 ± 2.49	7.72 (1.40, 14.04)	0.017	0.082
Non-dominant-leg Stance (s)	7.3 ± 12.7	16.6 ± 15.52	6.36 ± 2.61	17.09 ± 2.53	10.73 (4.36, 17.11)	0.005	0.144
Balance Beam Walk (s)	−1.9 ± 3.91	−2.6 ± 4.15	−1.46 ± 0.23	−2.62 ± 0.22	−1.15 (−1.72, −0.58)	<0.001	0.196
CoP Path Length (cm)	9.2 ± 23.59	−2.6 ± 32.62	12.71 ± 4.61	1.93 ± 4.4	−10.78 (−22.41, 0.84)	0.068	0.049
CoP AP Path Length (cm)	5.3 ± 13.6	−0.5 ± 21.88	5.25 ± 3.11	−0.58 ± 2.96	−5.84 (−13.69, 2.02)	0.286	0.032
CoP ML Path Length (cm)	11.7 ± 21.87	−3.2 ± 20.51	15.09 ± 3.66	0.87 ± 3.5	−14.22 (−23.42, −5.03)	0.012	0.124
CoP Sway Velocity (cm/s)	1.2 ± 2.16	0.4 ± 1.73	1.34 ± 0.36	0.54 ± 0.35	−0.80 (−1.70, 0.09)	0.231	0.046
CoP Sway Area (cm^2^)	0.9 ± 13.05	1.8 ± 9.61	2.45 ± 1.85	2.31 ± 1.78	−0.14 (−4.68, 4.4)	0.95	<0.001
Grip strength (kg)	0.5 ± 2.08	−0.3 ± 1.88	0.22 ± 0.4	−0.15 ± 0.39	−0.37 (−1.36, 0.62)	0.687	0.008
Sit and Reach (cm)	0.1 ± 5.02	0.9 ± 4.04	0.61 ± 0.63	1.11 ± 0.61	0.50 (−1.05, 2.06)	0.687	0.006
SLJ (cm)	1.9 ± 7.26	7.3 ± 7.14	0.66 ± 1.28	7.64 ± 1.23	6.98 (3.82, 10.15)	0.009	0.225
CMJ (cm)	4.2 ± 4.10	6 ± 5.05	3.83 ± 0.64	5.45 ± 0.62	1.62 (0.03, 3.21)	0.101	0.058
Double-leg Continuous Jump (s)	−0.2 ± 1.13	−1.4 ± 1.15	−0.16 ± 0.13	−1.23 ± 0.12	−1.06 (−1.37, −0.76)	0.009	0.416
15 m Zigzag Run (s)	−0.3 ± 0.52	−0.9 ± 0.41	−0.28 ± 0.09	−0.94 ± 0.09	−0.66 (−0.88, −0.44)	0.009	0.345
Height (cm)	1.0 ± 0.48	1.2 ± 0.69	0.97 ± 0.13	1.18 ± 0.12	0.21 (−0.10, 0.52)	0.32	0.027
Body mass (kg)	1.3 ± 0.77	1.3 ± 0.76	1.34 ± 0.16	1.36 ± 0.16	0.03 (−0.37, 0.43)	0.884	0.000
BMI (kg/m^2^)	0.7 ± 0.63	0.6 ± 0.5	0.7 ± 0.12	0.64 ± 0.11	−0.06 (−0.35, 0.23)	0.769	0.002

^a^: Mean ± SD; ^b^: Mean ± SE; ^c^: ANCOVA adjusted for baseline, class, age and sex. ^d^: *p*-values for the three key primary outcomes are unadjusted, for the five derived primary were adjusted by the Holm–Bonferroni procedure, and for the nine secondary outcomes were adjusted by the Benjamini–Hochberg FDR method. CI: Confidence Interval. LS: Least Squares. SD: Standard Deviation. SE: Standard Error. FDR: False Discovery Rate.

## Data Availability

The data is available on request from the corresponding author.

## Supplementary Materials

The following supporting information can be downloaded at: https://www.mdpi.com/article/10.3390/sports14070286/s1, File S1: CONSORT 2025 checklist; File S2: Basic movements included in the lower-limb muscular fitness-dominated kindergarten Wushu program; File S3: Correlation analysis results [31].

## Abbreviations

The following abbreviations are used in this manuscript:

AP	Antero-Posterior
ANCOVA	Analysis of Covariance
BMI	Body Mass Index
CMJ	Countermovement Jump
CON	Control
CoP	Center of Pressure
HRmax	Maximum Heart Rate
INT	Intervention
ML	Medio-Lateral
SLJ	Standing Long Jump
